# Barriers, motivations and physical activity among medical students: a comparative study between the University of Seville (Spain) and Paris-Saclay University (France)

**DOI:** 10.3389/fspor.2026.1795740

**Published:** 2026-06-29

**Authors:** Ángel-Tomás Parra-Martínez, Cecilio Parra-Martínez, Antonio Parralo-López, Sofía M Martínez-Sánchez, Raquel Martín-Riquel, Concha Martínez-García

**Affiliations:** 1 Orthopaedic Surgery and Traumatology, Virgen del Rocío University Hospital, Seville, Spain; 2 Doctoral School, Vice-Rectorate for Doctoral Studies, Language Policy and Library, University of Huelva, Huelva, Spain; 3 Département des Sciences de ĺEnvironnement et des Paléoenvironnements Océaniques et Continentaux, UMR 5805, Université de Bordeaux, Bordeaux, France; 4 Area of Medicine, University of Huelva, Huelva, Spain; 5 Department of Physiotherapy, Faculty of Health Sciences, EUNEIZ University of Vitoria, Vitoria-Gasteiz, Spain; 6 Area of Psychobiology, Department of Clinical and Experimental Psychology, Faculty of Ed., Psychology and Sports Sciences, University of Huelva, Huelva, Spain; 7 Area of Developmental Psychology, Faculty of Ed., Psychology and Sports Sciences, University of Huelva, Huelva, Spain

**Keywords:** barriers, health sciences, medical students, motivation, physical activity, World Health Organization

## Abstract

**Introduction:**

Studies conducted among university students show non-compliance with the recommendations for healthy physical activity, while the factors that hinder or facilitate its practice remain insufficiently understood. The aim of this study is to examine barriers, motivations, and the degree of compliance with physical activity among medical students, comparing University of Seville, Spain (US) and Paris-Saclay University, France (UP-S) samples.

**Methods:**

This cross-sectional empirical study included 223 undergraduate medical students (M = 22.30 years, SD = 2.259), from US and UP-S, 68.8% were women. In the selected classroom and in their respective languages, participants completed the instruments: International Physical Activity Questionnaire (IPAQ); habitual distances travelled; Self-Report of Barriers to Physical Exercise Practice (ABPEF); Goal Content for Exercise Questionnaire (GCEQ). The non-parametric Mann–Whitney U test was used, with significance set at *p* < 0.05, and correlational analyses were conducted using Kendall's tau-b (Tb) statistic in SPSS version 25.0.

**Results:**

UP-S students reported higher barriers than US students across all ABPEF factors, with significant differences in Total score (U = 4.616,5; *p* = 0.001), Fatigue/Laziness (*p* < 0.001), and Environment/Facilities (*p* = 0.002). US students showed significantly higher motivation in the Social Recognition factor of the GCEQ (*p* = 0.018). According to the IPAQ classification of three physical activity levels, US students were classified in the high physical activity level, whereas UP-S students were classified in the low and moderate levels (*p* < 0.001). The University/Country variable was associated with the distance from home to leisure facilities (*p* = 0.008), with the Total, Fatigue/Laziness, and Environment/Facilities (*p* = 0.002) barrier scores, and also with the Social Recognition motivational scale (*p* = 0.018).

**Discussion:**

The differences identified in our results highlight the need to consider the environmental context in studies on the physical activity of professionals directly involved in public health. US and UP-S medical students perceive Lack of time, primarily due to the high academic demands, as the main barrier to physical activity. Universities and European Higher Education Area policies should make greater efforts to provide physical and curricular environments that facilitate and motivate physical activity practice, within medical degree programs, a population that is key to the prevention of non-communicable diseases, in line with “Healthy Doctor – Healthy Patient”, and the 3rd Sustainable Development Goal.

## 1 Introduction

It is increasingly recognised that physical activity (PA) provides benefits for our overall bio-psycho-social health and for the prevention of chronic non-communicable diseases (NCDs), which are derived from unhealthy behaviours and habits such as sedentary lifestyles and increasing physical inactivity. Evidence has shown associations between physical inactivity and cardiovascular diseases (the leading cause of mortality worldwide), obesity, breast and colon cancer, osteoporosis, coronary heart disease and hypertension and dementia, among others (1–8). Similarly, regular engagement in PA has psychological benefits, reducing anxiety, stress, and depression, improving cognitive abilities, social skills, and self-concept, providing emotional stability, enhancing resilience, and preventing eating disorders (9–14).

The World Health Organization (WHO) (15) reports that NCDs in the European Union are responsible for 90% of all deaths, with PA being essential for their prevention. It is estimated that increasing PA, even to the minimum recommended levels, could prevent 11.5 million new cases of NCDs by 2050. In this context, the WHO launched the Global Action Plan on Physical Activity 2018–2030 (16), which includes 20 recommendations and concrete measures aimed at reducing physical inactivity by 15% by 2030 through the reorientation of habits towards healthier behaviours.

This Plan seeks to promote the comprehensive adoption of a response involving all sectors and stakeholders (countries, cities, and communities) in order to provide safe and supportive environments that help individuals increase their levels of physical activity. Among these recommendations are those established for adults aged 18–64 years, which include performing 150–300 min of moderate-intensity aerobic physical activity per week, or 75–150 min of vigorous-intensity aerobic physical activity, or an equivalent combination of both (16).

Therefore, one of the major current challenges in public health is reducing insufficient physical activity among young adults, as it is estimated that between 20% and 50% of young people worldwide do not achieve the recommended levels of physical activity (17).

Studies conducted in this age group have mainly focused on university students from different academic disciplines (18–20) and, in general, they have been described as a particularly vulnerable group. This is due, among other reasons, to academic demands and lifestyle changes associated with entering university, which often lead to the abandonment of adequate nutrition and regular physical activity previously maintained (only between 25% and 40% of university students engage in healthy levels of physical activity), shifting instead towards more sedentary patterns and higher consumption of unhealthy caloric intake such as unsaturated fats, and foods rich in sugars and salt (21–23).

Among the studies analysing motivations that may favour the practice of PA among university students and that also consider the cultural context through comparative analyses between different countries, the recent study by Galeano-Rojas et al. (20) stands out. This study compared Spain and Ecuador using the Goal Content for Exercise Questionnaire (GCEQ). The results showed significant differences in motives related to health improvement and skill development, both being more highly valued by Spanish students (M = 5.86 vs. M = 5.6, and M = 5.26 vs. M = 5.06, respectively), whereas social recognition as a motivation or goal was significantly more highly valued by Ecuadorian students (M = 3.82 vs. M = 2.98). Both groups of students did not differ (*p* > 0.05) in social affiliation or image-related motivations.

Focusing specifically on research conducted among medical students, who are focused on health and will become key influencers in the population once they begin practicing as physicians, the review by Sousa et al. (24) revealed various perceived barriers to practising PA and above all, gaps in related knowledge and skills related to physical activity and dietary promotion. The authors found a generally low prevalence (37.9%) of patient counselling on physical activity in primary care (25), as well as significant variability in the extent to which physicians perform screening for PA levels (2.4%–100%) and provide PA counselling (0.6%–100%). These practices were more frequent among patients with higher body mass index, lower levels of physical activity, and/or greater comorbidities (26). This highlights the need for more comprehensive and compulsory university training related to PA and nutrition (24).

The recent study conducted at the University of the Nile, Sudan (27) concluded that medical students understand and support the promotion of an active lifestyle (82.98%); however, this did not translate into an increase in regular practice (51.60% inactive), revealing the gap between knowledge and practice in adherence to healthy habits. In the European Union (EU), the study carried out in the Western Balkans (28) found differences in the proportion of students engaging in PA depending on the country and faculty, with lower levels of PA (50.9%) being associated with barriers such as low socio-economic status, overweight, and female sex.

Regarding motivations for PA among medical students (29) the study conducted in 14 faculties across five Balkan countries by Ilić et al. (30) identified the following motivators, in order of priority for engaging in physical activity (PA): feeling better, reducing stress, improving physical appearance, losing weight, and managing chronic diseases.

The objective of this study is to examine the main barriers and motivations for engaging in healthy PA in a sample of medical students from Spain and France, as well as to assess their physical activity levels based on WHO recommendations and everyday walking distances, and finally, to analyse whether relationships exist among these variables. To our knowledge, this is the first study conducted between these two neighbouring European Union (EU) countries to comprehensively investigate these health-related influencing factors.

## 2 Method

### 2.1 Study design, setting, and participants

This was a comparative cross-sectional study with non-probability convenience or purposive sampling. Participant recruitment and data collection were carried out during the 2023/2024 academic year at medical schools of University of Seville (US) in Spain, and Université Paris-Saclay (U P-S) in France. During the previous academic year, the necessary organisational and inter-university coordination was carried out. The questionnaires, in the students' native language, were administered in person at each university, in the classroom where students had scheduled lessons with the agreed teaching staff.

Inclusion criteria were: being between 18 and 64 years of age (WHO age range in its recommendations), being enrolled in the 4th or 5th year of the Medicine Degree at one of the two participating universities (close to completion of studies), absence of diseases preventing the practice of physical exercise, and signing the informed consent form. Exclusion criteria were failure to meet any of the inclusion criteria.

For the initial calculation of sample size, the 2022/2023 graduation rate of medical students at the US was considered, resulting in 256 graduates. An attendance rate of 80% was estimated (205 students). Statistical power was set at.95 (95%) to apply the following formula (Cochran's formula): Sample size = Z² × p × (1−p)/c², where Z represents the 95% confidence level, *p* = 0.5, and c is the margin of error 4%.

This calculation yielded a result of 135 for the US sample. For the estimation of the sample size of medical students at UP-S, the number of first-year places (267 newly enrolled students) was used, as the graduation rate was not available. The same parameters as those applied to the US sample were used, assuming an attendance rate of 80% (214 students). This resulted in a required sample size of 138 students for the UP-S cohort, which was similar to that estimated for the US cohort (135). This led to an expected total sample of 273 participants. Nevertheless, the final number of participants corresponded to those attending class on the day of data collection and therefore did not depend strictly on the number of enrolled or graduated students.

Finally, the study sample consisted of 223 medical students (US = 113 and UP-S = 110) who attended class on the day, all of whom freely and voluntarily agreed to participate in the study (see Table 1).

**Table 1:** Sociodemographic results.

Variables	Total	U.P-S	US
N	223	110	113
Age: M (SD)	22.35 (2.202)	21.86 (1.417)	22.83 (2.679)
Sex: N (%)			
Woman	149 (66.8)	81 (73.6)	68 (60.2)
Man	74 (33.2)	29 (26.4)	45 (39.8)
Academic year: N (%)			
4th	107 (48.0)	93 (84.5)	14 (12.4)
5th	116 (52.0)	17 (15.5)	99 (87.6)
Distance from home to gym: N (%)			
< 2 km	154 (69.1)	73 (66.4)	81 (71.7)
2–4 km	42 (18.8)	21 (19.1)	21 (18.6)
5–8 km	11 (4.9)	8 (7,3)	3 (2.7)
> 8 km	16 (7.2)	8 (7.3)	8 (7.1)
Distance from home to university: N (%)			
< 2 km	85 (38.1)	50 (45.5)	35 (31.0)
2–4 km	47 (21.1)	12 (10.9)	35 (31.0)
5–8 km	30 (13.5)	15 (13.6)	15 (13.3)
> 8 km	61 (27.4)	33 (30.0)	28 (24.8)
Distance from home to recreational area: N (%)			
< 2 km	76 (34.1)	33 (30.0)	43 (38.1)
2–4 km	64 (28.7)	22 (20.0)	42 (37.2)
5–8 km	38 (17.0)	28 (25.5)	10 (8.8)
> 8 km	45 (20.2)	27 (24.5)	18 (15.9)

U P-S, University París-Sanclay; US, University of Seville.

### 2.2 Measures and instruments

The study variables and instruments used for their assessment were:

-Sociodemographic data: university/country, age, sex, and year of study.

-Habitual distances (*ad hoc* instrument): a questionnaire specifically designed for this study with three items on the distance from home to usual destinations: sports facility, university/hospital, and leisure venue. Each item offered four response options: <2 km; 2–4 km; 5–8 km; and >8 km.

-PA performed during the last 7 days: IPAQ (International Physical Activity Questionnaire), validated in Spanish by Román-Viñas et al. (31) The short version consists of 7 items and provides information on the time spent performing moderate- and vigorous-intensity activities, walking, and sitting. The IPAQ allows individuals to be classified into three categories (low, moderate, high) according to the estimated energy expenditure for each activity: vigorous, 8 METs (Metabolic Equivalent of Task); moderate, 4 METs; and walking, 3.3 METs. It also enables assessment of compliance with PA recommendations (30 min of moderate PA per day), categorizing individuals as compliant (high or moderate IPAQ) or non-compliant (low IPAQ). The instrument shows high inter-observer reliability, ICC = 0.91 (Intraclass Correlation Coefficient) and adequate internal consistency for each of its dimensions (*α*= 0.81–0.88).

-Barriers to physical exercise practice: ABPEF (Spanish acronym of Self-Report of Barriers to Physical Exercise Practice) by Niñerola et al. (32) Consists of 17 items with Likert scale from 0 to 10 (Likert 0–10) divided into four factors: Body Image/Social Physical Anxiety, Fatigue/Laziness, Obligations/Lack of Time, and Environment/Facilities. The ABPEF shows high internal consistency (*α*=.86) and significant correlations in the exploratory factor analysis across four factors (33).

-Motivations or goals for PA practice: GCEQ (Goal Content for Exercise Questionnaire), in its Spanish-validated version by Sicilia et al. (34) consisting of 20 items (Likert 1–7) associated with five factors: social affiliation, health, skill development, image, and social recognition. The internal consistency of the scale factors yielded *α* values ranging from.70 to.89.

### 2.3 Procedure

Participants were contacted in person during one of their regular classes in each country, with prior agreement from the teaching staff responsible for the sessions in each case.

Previous collaborative contacts had already been established between both universities, and the following information was provided to facilitate its dissemination among students in their native language:

The study aims to analyse certain characteristics that hinder or facilitate the level of physical activity and its relationship with the self-perception of barriers and motivations to engage in PA in two international samples of medical students from Spain and France. This population is particularly relevant because they will be responsible for prescribing the World Health Organization (WHO) healthy lifestyle recommendations in this field in their future professional practice.

In the instruments (questionnaires/scales) that participants were asked to complete, there were no right or wrong or true or false answers; rather, what matters is each participant's own perception of the situations presented. Therefore, sincerity in responding was considered essential. Responses were anonymous and were used solely for research purposes by the researchers of the responsible university (UHU), with only statistical data being reported and no individual data disclosed. The ultimate aim of the study is to contribute to improving healthy physical activity behaviours. Participation was voluntary, and participants were free to withdraw at any time (the Informed Consent document was provided).

The requirements for participation in the study were:

1. To be over 18 years of age.
2. To be a 4th- and/or 5th-year medical student at one of the two participating universities.
3. Not to suffer from any disease that prevents the practice of physical exercise.
4. To sign the Informed Consent form.

On the agreed date for data collection, the principal investigator provided an explanatory reading of the Informed Consent (IC) document and distributed the booklets containing the instruments and the IC form for signature among those who voluntarily agreed to participate and met the inclusion criteria.

All students present in the classroom participated and constituted the study samples for each country.

### 2.4 Statistical analyses

Initially, the Kolmogorov–Smirnov test was used to assess data normality. Subsequently, as the assumption of normality was not met, the non-parametric Mann–Whitney U test (U M-W) was applied to compare the mean between two independent groups. In this study, the independent variable was the university/country, and the dependent variables were the scores obtained for the items, factors, and total of the questionnaires on distances, IPAQ, ABPEF, GCEQ, and age.

Descriptive statistical analyses were performed, obtaining absolute frequencies (n), relative frequencies (%), measures of central tendency (M), and dispersion (SD). The hypothesis of equality of means (*p* < 0.05) was tested using Chi-square (*χ*²) to compare qualitative variables. Subsequently, the U M–W (U) was applied to all dependent variables, comparing students from Spain and France. Effect sizes (Cohen's d/Cramer's V) and *post hoc* statistical power (95% CI) were also calculated using total n, *α* = 0.05, two-tailed test, interpreted as small (±0.20), medium (±0.50), and large (±0.80) effects for UP-S vs. US comparisons. Finally, Kendall's tau-b (Tb) statistic was used to analyse the correlation coefficients between the variables and ordinal regression analysis with a logit link (cumulative proportional odds model) was performed based on the significant correlations obtained through the tau-b coefficient. The dependent variable in the model was the IPAQ PA Level, while the predictor variables were those that showed significant correlations with it. The cumulative logit model (cumulative proportional odds) was assessed using the test of parallel lines, with the assumption being satisfied. Multicollinearity was ruled out, as variance inflation factor (VIF) values were below 5 for all predictors. Statistical analyses were conducted using SPSS Statistics for Windows, version 25.0.

### 2.5 Ethical approval

The study protected participants' anonymity and confidentiality through an alphanumeric code (Organic Law 3/2018 of 5 December on the Protection of Personal Data and the Guarantee of Digital Rights), adhered to the Declaration of Helsinki of October 2013, and to Law 41/2002 of 14 November, as amended on 1 March 2023. The study was approved by the Regional Biomedical Research Ethics Committee under code: SICEIA-2024-002968 and study code: MOT-AF-MED-2024 (SICEIA: Spanish acronym of Information System of the Research Ethics Committees of Andalusia).

## 3 Results

### 3.1 Sample characteristics

A total of 223 medical students from the 4th and 5th years, enrolled at universities in Spain (US) and France (UP-S), participated in the study. Their mean age was 22.35 years (SD = 2.202), with women accounting for 66.8%. Table 1 presents the summary of sociodemographic results for both universities/countries. In which a statistically significant difference was observed in the academic year distribution between the UP-S and US cohorts (*χ*²= 116.273; *p* < 0.001), with the vast majority of UP-S students being in the fourth year of Medicine, whereas most US students were in the fifth year. Additionally, the sex distribution differed significantly between groups (*χ*²= 4.554; *p* = 0.033), with women outnumbering men in both samples, and a higher proportion of women in the UP-S sample compared to the US sample.

The reported habitual daily distances in both samples were similar regarding the distance to the sports facility (*χ*²= 2.648; *p* = 0.449). However, significant differences were observed in the distance to the university (*χ*²= 14.274; *p* = 0.003), with UP-S students more frequently reporting distances of <2 km and >8 km, while US students reported <2 km and 2–4 km. Distances to leisure venues also differed (*χ*²= 17.855; *p* < 0.001), as UP-S students were more evenly distributed across the four available distance ranges, whereas US students reported <2 km and 2–4 km.

The effect size (d/V) and approximate power analysis (d → 95% CI) for each distance variable were interpreted as follows: for sports facilities, differences between universities/countries were minimal (V = 0.11, small effect; 95% CI 0.00–0.22, low); distance to the university (V = 0.25) showed a small-to-medium effect size and high power (95% CI 0.14–0.36); and distance to leisure locations showed clearer differences between both universities/countries with a medium effect (V = 0.28) and very high power (95% CI 0.17–0.39), with US students having leisure locations closer to their residence.

The significant differences found in daily travel distances between universities/countries using this *ad hoc* instrument suggest that shorter distances in Spain are more likely to be covered on foot or by bicycle, thus involving physical activity, whereas longer distances in France suggest a higher probability of using urban transport, which would result in lower daily physical activity levels. Nevertheless, these findings should be interpreted with caution, as they represent a contextual exploratory indicator.

### 3.2 Physical activity performed during the last 7 days (IPAQ)

Table 2 presents the mean values and standard deviations of students from both universities/countries derived from the administration and analysis of the IPAQ, together with the statistical significance obtained using the U M-W across all PA levels factors, as well as effect size and statistical power (95% CI). The results were significantly different between US and UP-S medical students in all IPAQ items (weekly and minutes/day), with the US students being significantly more physically active.

**Table 2:** Results for French (UP-S) and Spanish (US) medical students on the IPAQ and statistical comparison of differences (p) using the non-parametric Mann–Whitney U test (U M–W).

IPAQ	U.P-S (*n* = 110)	US (*n* = 113)	U M-W (p)	d	Interpr.	(95% CI)
Intense Physical Activity						
Weekly (days)	1.67 ± 1.828	2.82 ± 1.809	<0.001	0.63	Medium-High	0.37–0.89
Minutes per day	50.64 ± 54.171	64.20 ± 42.229	0.005	0.28	Low	0.02–0.54
Moderate Physical Activity						
Weekly (days)	1.14 ± 1.820	1.96 ± 1.975	<0.001	0.43	Medium	0.17–0.69
Minutes per day	27.41 ± 40.883	44.16 ± 38.729	<0.001	0.42	Medium	0.16–0.68
Walking/Light Physical Activity						
Weekly (days)	5.99 ± 1.753	6.38 ± 1.352	0.044	0.25	Low	−0.01–0.51
Minutes per day	53.36 ± 46.655	72.62 ± 54.609	0.003	0.38	Low-Medium	0.12–0.64
Seated						
Minutes per weekday	545.09 ± 104.344	498.14 ± 152.680	0.006	0.36	Low-Medium	0.10–0.62
Total PA Level						
	2.35 ± 0.672	2.70 ± 0.565	<0.001	0.56	Medium	0.30–0.82

Interpretative analysis of effect size (d) and statistical power (95% CI).

Descriptive values are presented as Mean ± Standard Deviation. IPAQ, international physical activity questionnaires; U P-S, University París-Saclay; US, University of Seville. *d*: effect size (Cohen's *d*); (95% CI), 95% confidence intervals; Interpr., interpretation of the effect–power association.

Interestingly, UP-S students only showed higher scores in the sitting item (more minutes per working day), which was significantly different compared to US students, who spent less time sitting.

The detailed analysis of effect size and confidence intervals (Table 2) regarding time spent by both samples on each type of IPAQ activity showed clearer differences in:

- Days of vigorous activity (d = 0.63, 95% CI 0.37–0.89)
- Total physical activity level (d = 0.56, 95% CI 0.30–0.82)

Students from Seville devoted more days and minutes to vigorous activity, moderate activity, and walking, and spent less time sitting. Most effects were medium or small, but with adequate statistical power (95% CI), indicating that the observed differences are real and not artefacts of insufficient sample size.

#### 3.2.1 Physical activity level classification

**Figure 1:**
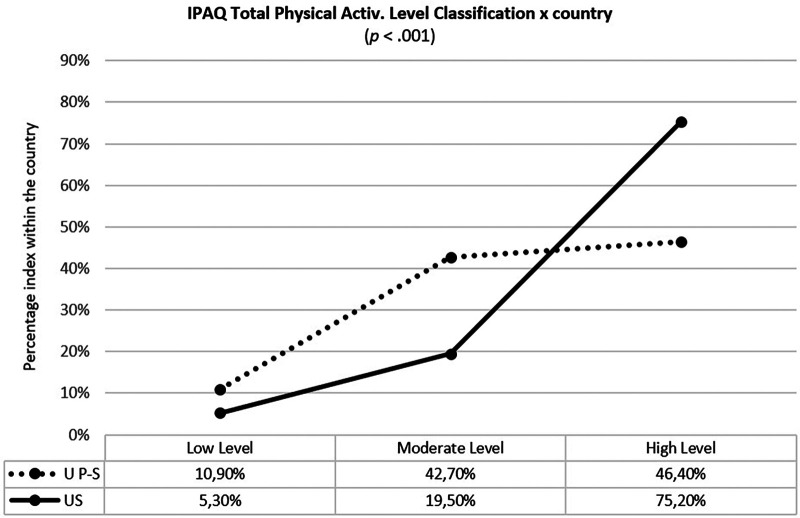
Physical activity classification level by IPAQ of Spanish (US) and French (U P-S) medical students. Across the three IPAQ activity levels, students from both countries differed significantly in their classification (*p* = 0.000): French students were mostly classified as Low or Moderate, while Spanish students were predominantly classified as High PA level. IPAQ: International Physical Activity Questionnaire; U P-S: University Paris-Sanclay; US: University of Seville.

The results derived from the IPAQ in the classification of Low, Moderate, and High PA are presented in Figure 1. The analyses revealed significant differences between medical students from both universities/countries (U = 7,999.5; *p* < 0.001), with UP-S students classified at Low and Moderate PA levels, whereas US students were classified at the High PA level. Effect size and power analysis of IPAQ results showed the most marked difference between both samples, with a medium-to-large effect size (V = 0.30; 95% CI 0.30–0.82). With a sample size of *n* = 223 and this effect magnitude, the probability of detecting the association is very high. The US sample showed a higher percentage of students at high physical activity levels (75.20% vs. 46.40%), while the French sample showed a higher percentage at low levels (10.90% vs. 5.30%) (see Figure 1).

### 3.3 Barriers to physical activity (ABPEF)

The barriers reported by UP-S and US medical students are shown in Table 3, where UP-S students perceived greater barriers than US students in 15/17 items of the ABPEF. The results across the five scales of the questionnaire can be seen in Table 4. Across all scales measuring perceived barriers to physical activity, UP-S students displayed higher mean scores, with significant differences observed in Fatigue/Laziness, Environment/Facilities, and the ABPEF Total.

**Table 3:** Results of responses to the items and scales of the ABPEF, of French (UP-S) and Spanish (US) medical students, and statistical comparison of the differences (*p*) with the non-parametric U Mann–Whitney test.

ABPEF “The reason that prevents me from performing physical exercise during the upcoming weeks”		U P-S (*n* = 110)	US (*n* = 113)	U M-W (p)
Body Image/Physical social anxiety				
3	‘Feeling uncomfortable about the way I look in sports clothes.’	2.22 ± 2.215	2.10 ± 2.175	0.768
6	‘Feeling that my physical appearance is worse than that of others'	1.99 ± 1.993	2.39 ± 2.339	0.147
10	‘Thinking that other people are in better shape than me'	2.43 ± 2.297	2.22 ± 2.325	0.196
13	‘Thinking that others judge my physical appearance’	7.91 ± 2.410	7.33 ± 2.737	0.785
16	‘Feeling embarrassed because they are watching me while I exercise’	2.73 ± 2.523	2.02 ± 2.048	0.016
Fatigue/Laziness				
1	‘Getting too tired during exercise or being afraid of getting injured’	4.83 ± 2.749	2.73 ± 2.304	<0.001
2	‘Being lazy’	6.33 ± 2.899	5.42 ± 2.962	0.021
5	‘Having sore muscles or pain as a result of exercise’	3.78 ± 2.610	3.15 ± 2.338	0.066
8	‘Not being “in shape” to exercise’	4.28 ± 2.740	2.72 ± 2.422	<0.001
9	‘Unwillingness to be constant’	5.56 ± 3.221	4.18 ± 2.989	0.001
12	‘Notice tiredness or fatigue regularly throughout the day’	5.09 ± 2.985	3.96 ± 2.763	0.004
Obligations/Lack of time				
4	‘Having too much work’	8.47 ± 1.948	8.01 ± 2.339	0.147
7	‘Having too many family obligations'	7.84 ± 2.336	8.09 ± 2.441	0.189
11	‘Not finding the time necessary for exercise’	7.91 ± 2.410	7.33 ± 2.737	0.104
Environment/Facilities				
14	‘Being too far from where I can exercise’	3.21 ± 2.480	2.53 ± 2.361	0.011
15	‘Being uncomfortable with people who exercise with me’	1.68 ± 1.597	1.87 ± 1.698	0.448
17	‘The facilities or monitors are not adequate’	3.21 ± 2.563	2.03 ± 1.962	<0.001
Total ABPEF score				
Total ABPEF score		4.51 ± 1.295	3.94 ± 1.357	<0.001

Descriptive values are presented as Mean ± Standard Deviation. ABPEF, self-report of barriers to the practice of physical exercise; U P-S, University Paris-Sanclay; US, University of Seville.

**Table 4:** Results of French (UP-S) and Spanish (US) medical students' responses to the ABPEF scales; statistical comparison of differences using the non-parametric Mann–Whitney U test, with an interpretative analysis of effect size and statistical power (95% CI).

ABPEF: Self-Report of Barriers to the Practice of Physical Exercise	Country (Univ.)	N	Mean	Standard Deviation	U M-W (p)	d	Interpr.	(95% CI)
Body Image/ Physical social anxiety	U P-S	110	2.2782	1.7676	U = 5.790,5;	0.09	Low	−0.17–0.35-
	US	113	2.1239	1.7756	*p* = 0.362			
Fatigue/Laziness	U P-S	110	4.9788	2.0453	U = 3.996,0;	0.65	Medium-High	0.39–0.91
	US	113	3.6917	1.8998	*p* < 0.001			
Obligations/ Lack of time	U P-S	110	8.0727	1.9222	U = 6.009,5;	0.12	Low	−0.14–0.38
	US	113	7.8083	2.2951	*p* = 0.668			
Environment/ Facilities	U P-S	110	2.7000	1.6730	U = 4.737,5;	0.34	Low-Medium	0.08–0.60
	US	113	2.1416	1.6102	*p* = 0.002			
Total ABPEF score	U P-S	110	4.5067	1.2948	U = 4.616,5;	0.43	Medium	0.17–0.69
	US	113	3.9414	1.3568	*p* = 0.001			

U P-S, University Paris-Saclay; US, University of Seville; *d*, effect size (Cohen's *d*); (95% CI), 95% confidence intervals; Interpr, interpretation of the effect size–power association.

Effect sizes (Cohen's d) and 95% confidence intervals (95% CI) were calculated for UP-S vs. US comparisons across the five ABPEF scales. Notably, a medium-to-large effect (d = 0.65, 95% CI 0.39–0.91) was observed in the Fatigue/Laziness scale, with UP-S students reporting higher fatigue or lack of motivation as a barrier, as well as a medium effect in the total ABPEF score (d = 0.43, 95% CI 0.17–0.69). The remaining barriers reported by participants were practically equivalent between the two samples of medical students.

### 3.4 Motivations or objectives for physical activity (GCEQ)

**Figure 2:**
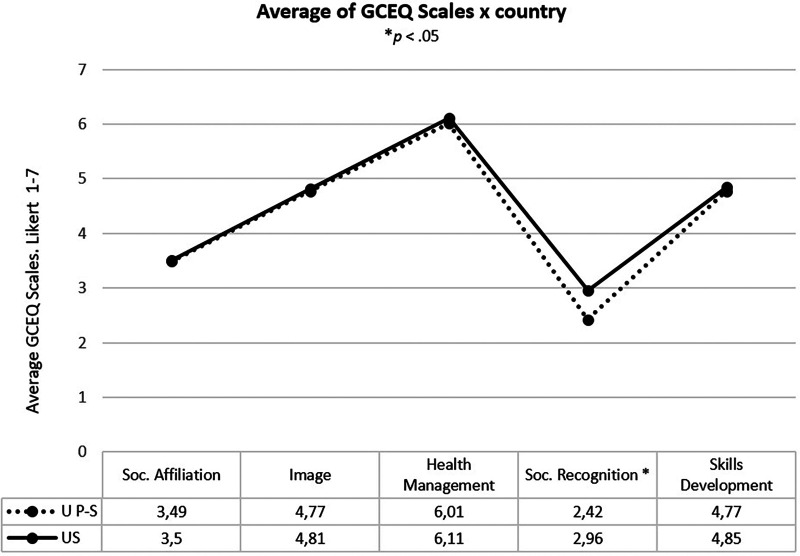
Mean scores on the scales of motivations or goals for physical activity (GCEQ) among Spanish (US) and French (U P-S) medical students. Spanish medical students assigned significantly greater importance than French students to the GCEQ Social Recognition scale (*p* = 0.018). Scores on the remaining scales were also lower of French students, although these differences were not statistically significant. GCEQ: Goal Content for Exercise Questionnaire; U P-S: University Paris-Sanclay; US: University of Seville.

Similar to the results presented regarding barriers to PA, Table 5 contains the data corresponding to the motivations or goals that facilitate PA (GCEQ items) reported by medical students from both universities/countries. US medical students assigned greater importance than UP-S students to 3/4 items on Social Recognition (U = 7,351.0; *p* = 0.018), as well as to the Health item related to increasing resistance to disease (*p* = 0.004). No significant differences were found in the other GCEQ scales, although UP-S students' scores were lower, indicating somewhat lower general motivation to engage in physical activity (see Figure 2).

**Table 5:** Results of responses to the items and scales of the GCEQ among French (UP-S) and Spanish (US) medical students, and statistical comparison of differences (*p*) using the non-parametric Mann–Whitney U test.

GCEQ ‘The reason that motivates me to exercise during the next few weeks'		U P-S (*n* = 110)	US (*n* = 113)	U M-W (p)
Social Affiliation				
1	To connect with others in a meaningful manner	3.67 ± 1.853	3.75 ± 1.724	0.843
6	To share my exercise experiences with people that care for me	3.85 ± 1.798	3.83 ± 1.812	0.966
11	To develop close friendships	3.20 ± 1.647	3.30 ± 1.747	0.685
16	To form close bonds with others	3.25 ± 1.741	3.10 ± 1.768	0.460
Image				
2	To improve the look of my overall body shape	5.58 ± 1.252	5.58 ± 1.193	0.859
7	To improve my appearance	5.37 ± 1.445	5.52 ± 1.296	0.631
12	To be slim so to look attractive to others	3.79 ± 1.877	3.73 ± 1.885	0.808
17	To change my appearance by altering a specific area of my body	4.34 ± 1.888	4.40 ± 1.595	0.966
Health Management				
3	To increase my resistance to illness and disease	5.45 ± 1.536	6.04 ± 1.038	0.004
8	To increase my energy level	6.17 ± 0.985	5.95 ± 1.187	0.206
13	To improve my overall health	6.45 ± 0.774	6.51 ± 0.814	0.342
18	To improve my endurance, stamina	5.96 ± 0.957	5.96 ± 1.133	0.560
Social Recognition				
4	To be well thought of by others	2.88 ± 1.652	3.46 ± 1.861	0.022
9	To be socially respected by others	2.39 ± 1.421	2.74 ± 1.797	0.265
14	To gain favourable approval from others	2.09 ± 1.310	2.79 ± 1.920	0.021
19	So that others recognize me as an exerciser	2.33 ± 1.472	2.86 ± 1.807	0.039
Skills Development				
5	To acquire new exercise skills	5.51 ± 1.332	5.35 ± 1.450	0.414
10	To learn and exercise new techniques	4.81 ± 1.705	4.83 ± 1.658	0.996
15	To become skilled at a certain exercise or activity	3.57 ± 1.855	3.99 ± 1.820	0.093
20	To develop my exercise skills	5.18 ± 1.540	5.23 ± 1.558	0.803

Descriptive values are presented as Mean ± Standard Deviation. GCEQ, goal content for exercise questionnaire, U P-S, University París-Sanclay; US, University of Seville.

In the GCEQ, differences between France and Spain showed very small or negligible effect sizes in most scales, except for Social Recognition, which presented a small-to-medium effect (d = 0.38, 95% CI 0.12–0.64), consistent with the observed statistical significance.

### 3.5 Correlations between variables

The correlational analysis of the study variables using Kendall's tau-b (Supplementary Table S1) yielded the following significant associations.

The variable University/Country was associated with the distance from home to leisure venues, indicating that UP-S students reported longer distances; with the level of PA in the IPAQ, showing that US medical students engaged in PA with greater intensity than French students; it also showed correlation with the ABPEF barriers Fatigue/Laziness, Environment/Facilities, and Total, demonstrating that these represented greater barriers for UP-S students than for US students; and finally, a significant correlation was also observed between university/country and the motivational scale Social Recognition (GCEQ), with this desire being more highly valued by US students (see Supplementary Table S1).

An ordinal logistic regression model with a cumulative logit link (cumulative proportional odds model) was conducted. Predictors were selected based on significant bivariate associations with IPAQ physical activity level, as assessed by Kendall's tau-b, alongside their theoretical relevance. These included university/country, gender, fatigue/laziness, obligations/lack of time, social recognition, skill development, and academic year. Age was additionally included as a covariate to control for potential confounding effects.

The IPAQ physical activity level was the dependent variable, with country, gender, fatigue/laziness, obligations/lack of time, social recognition, skills development, age, and academic year as predictors. The estimated coefficients (*β*) represent the change in the logit of the cumulative probability of being in a higher IPAQ category. These coefficients, together with their standard errors (SE), odds ratios (OR), and *p*-values, are presented in Supplementary Table S2.

The model showed a significant fit (*χ*²= 34.82, *p* < 0.001), and odds ratios (OR) with 95% confidence intervals were reported. The proportional odds assumption was assessed using the test of parallel lines and was met *χ*²= 12.14, *p* = 0.41. Furthermore, multicollinearity was ruled out, with variance inflation factor (VIF) values below 5 for all predictors.

The estimated coefficients (*β*) represent the change in the logit of the cumulative probability of being in a higher IPAQ category for each unit increase in the predictor, while holding the remaining variables constant. For ease of interpretation, the coefficients were transformed into odds ratios (OR), such that OR > 1 indicates an increased likelihood of belonging to higher physical activity categories, whereas OR < 1 indicates a decreased likelihood.

The coefficients are consistent with the magnitude and direction of the tau-b correlations and are expressed as log-odds (*β*), standard errors (SE), odds ratios (OR), and *p*-values (Supplementary Table S2).

The interpretation of the coefficients (odds ratios) from the ordinal regression analysis predicting the IPAQ PA Level variable is summarised as follows:

- University/Country (1 = US) (OR = 2.27): Students from Seville are 2.27 times more likely to be in a higher IPAQ category compared with students in Paris.
- Gender (1 = male) (OR = 1.99): Men show nearly twice the likelihood of being in higher levels of physical activity than women.
- Academic Year (OR = 1.30): For each additional academic year, the likelihood of being in a higher IPAQ category increases by 30%.
- Fatigue/Laziness (OR = 0.41): Fatigue reduces the likelihood of being in a higher IPAQ category by 59% (1–0.41 = 0.59).
- Obligations/Lack of Time (OR = 0.54): Academic or personal obligations reduce the likelihood of achieving high levels of physical activity by 46%.
- Social Recognition (OR = 1.32): Each increase in this motivation raises the likelihood of being in a higher IPAQ category by 32%.
- Skills Development (OR = 1.67): This motivation increases the likelihood of being in higher levels of physical activity by 67%.
- Age (OR = 0.99): Age does not show a significant effect on physical activity level (*p* = 0.842).

The model confirms that physical activity levels depend on a combination of contextual factors (university/country), personal factors (gender), psychological and academic barriers (fatigue, lack of time), and social motivations.

## 4 Discussion

Under the Healthy Doctor - Healthy Patient (35) purpose, in this investigation into PA among US and UP-S medical students, we evaluated and analysed three major factors influencing compliance with WHO recommendations for healthy PA, and the prevention of NCDs in individuals aged 18–64 years. These were: (a) PA during the previous 7 days and distances to habitual destinations; (b) the perceived barriers hindering regular PA; and (c) the main motivations facilitating its practice. In addition, correlational analyses, as well as effect size and statistical power analyses, were conducted for these variables to examine the set of associations influencing this sample of European medical students. Our inferences from the results obtained are presented below, discussed in relation to previous studies.

Firstly, we consider it noteworthy in our study to have included a record of participants' daily distances measured from home to the university, to the gym, and to leisure areas, for two main reasons: first, because doing so immediately before administering the IPAQ would prompt them to take into account the PA they habitually perform, thereby increasing the accuracy of their responses in the IPAQ; second, to gain a certain degree of control over habitual PA within the environmental context of each university/country, anticipating that shorter distances, up to 4 km, would generally be covered on foot or by bicycle, whereas longer distances (5–8 km or more) would involve the use of public or private transport and result in less PA. The findings in this regard generally indicate that in Spain such distances were shorter than in France, and US students also proved to be more active in the IPAQ. Furthermore, the distance to the sports facility was significantly associated with the ABPEF barrier Environment/Facilities, indicating that the greater the distance to the gym, the more it was perceived as a barrier hindering PA practice. It also showed other associations with motivational variables.

The analysis of effect size and statistical power for distance variables showed a medium effect size and very high power (95% CI) for distance to leisure locations (*χ*² = 17.855; V = 0.28; 95% CI 0.17–0.39), and a small-to-medium effect size with high power for distance to the university (*χ*² = 14.274; V = 0.25; 95% CI 0.14–0.36). Therefore, these daily distances appear to be a key determinant of active behaviour, with differences between both international samples being robust and meaningful. These findings are directly related to the greater engagement in physical activity observed among US medical students, who accumulate more days and minutes of vigorous, moderate, and walking activity compared to their UP-S counterparts. It may be inferred that one of the possibly contributing factors underlying these results is the substantial investment in infrastructure for cycling lanes and pedestrian pathways in recent years in the Spanish city included in the study.

In any case, the findings obtained here, as a pilot study, suggest that this measurement of daily distances, created specifically for this study, is useful for complementing the estimation of habitual PA, although its validity still needs to be confirmed in future studies and these findings should be interpreted with caution, as they represent a contextual exploratory indicator.

Regarding PA during the previous 7 days (IPAQ), the results showed that at the Low and Moderate PA levels, UP-S students exceeded US students (10.9% and 42.7% vs. 5.3% and 19.5%, respectively). Conversely, at the High PA level, US students exceeded UP-S students (75.2% vs. 46.4%). When the total sample was grouped into two categories (active and inactive), considering active those with Moderate and High PA levels, 91.9% were classified as active (30.9% Moderate and 61% High). Therefore, these medical students comply with WHO recommendations. The results of the regression model confirm that this variable is influenced by psychological barriers, such as fatigue and lack of time due to academic obligations, as well as by contextual and social factors, which predict higher or lower levels of physical activity in both samples of medical students.

For the discussion of these findings in relation to the relevant literature, studies conducted in European populations using the IPAQ among medical students were taken as reference, since no previous studies of this type were found in Spain or France.

The study by Dąbrowska-Gałas et al. (36) conducted in Poland, revealed that 19% of medical students were inactive and approximately 81% met the WHO recommendations on PA, indicating a lower proportion of active students than those observed here. Fisher et al. (37) in Cyprus, found that the majority of participants (60.8%) reported a high level of physical activity and 32.9% a moderate level, resulting in 93.7% of students being active. These latter results are closer to, and even more favourable than, those observed in the present study; however, the sample of Fisher et al. (37) was smaller (*n* = 79) compared with our study (*n* = 223), conducted in a single university, and their students had just entered the first year of medical school, whereas here we analysed those closer to graduation, in the 4th and 5th years, considering that this level of PA may be closer to what they will subsequently prescribe/recommend to their patients. In this regard, we can affirm that the results of the European medical students in our study are more representative of compliance with WHO recommendations than those reported by Fisher et al. (37, 38). Nevertheless, continued efforts in this direction are necessary, in view of the differences observed between students from Spain and France, it would be advisable to reduce the proportion of moderate PA in favour of higher levels of PA, based on their youth and health-oriented vocation and, especially because doctors who engage in more physical activity are associated with dedicating more time advising their patients about exercise (38).

The present findings indicate that US medical students engage in higher levels of physical activity, particularly of vigorous intensity, compared to their UP-S counterparts. This difference may be partly explained by environmental factors, as favourable weather conditions—such as those typical of countries like Spain—are associated with greater engagement in outdoor physical activity (39). Additionally, shorter daily distances and greater accessibility to recreational and sports facilities in cities such as Seville may promote active commuting and regular exercise.

In contrast, urban contexts such as Paris, characterised by longer commuting distances and greater reliance on motorised transport, may limit opportunities for incidental physical activity and may contribute to more sedentary behaviours. This interpretation aligns with previous research demonstrating the strong influence of built environments on physical activity levels (40). Overall, both environmental and contextual factors appear to underpin the observed differences between these populations.

With regard to barriers and motivations for PA practice, UP-S medical students reported significantly greater barriers than their US counterparts in the Fatigue/Laziness, Environment/Facilities, and ABPEF Total scales. Both groups assigned the highest scores to the factor Obligations/Lack of time. However, the opposite pattern was observed in relation to motivations for PA practice (GCEQ), with US students showing higher averages, particularly in the desire for Social Recognition and for increasing resistance to disease. Notably, the higher levels of PA observed among US students may be partly influenced by the greater proportion of participants in more advanced academic years, as academic year emerged as a significant predictor, suggesting that senior students may be better able to organise their time and incorporate PA into their routines. No studies of this type in Spanish or French samples were found that would allow an adequate comparison in the present analysis. Therefore, following the previous comparative approach regarding PA levels, we consulted European studies on barriers and motivations for PA among medical students, identifying only the work of Ilić et al. (30) in the Western Balkans.

In that study, barriers related to academic obligations were the most common obstacle to regular PA among medical students, consistent with our findings. Their most common motivations for regular PA were the desire to feel better, reduce stress, lose weight, and manage chronic conditions. Although our results largely coincide in identifying lack of time due to academic obligations as the main barrier and the motivation to increase resistance to disease, the study by Ilić et al. (30) despite its large sample of European medical students, did not employ validated and reliable assessment instruments such as those used here (ABPEF and GCEQ). Instead, they designed a series of multiple-choice questions specifically for that study, administered online on a large scale. For this reason, a more exhaustive comparison between the two investigations cannot be made.

It may therefore be inferred that the previously described aspects related to climate, distances, infrastructure, and the differing demands of higher education curricula in both universities/countries may contribute to the variations in physical activity levels observed in our findings. Furthermore, this highlights a broader objective: the need to investigate these variables in an integrated manner, rather than in isolation, in order to achieve a more realistic understanding of the complexity of physical activity and its relationship with health.

### 4.1 Study strengths

This is the first study conducted among medical students from these two neighbouring EU countries, Spain and France, providing interrelated data on the physical activity performed, as well as the perceived barriers and motivations to increase PA levels. It offers an integrated perspective that poses challenges for university policies, health professionals, and the public health system.

An additional advantage is the contribution of the first results obtained through a simple instrument evaluating distances between the participants' usual locations. Because of their everyday nature, the physical activity involved in these habitual commutes often goes unnoticed. Although this should be considered a pilot study due to the lack of statistical studies on its reliability and validity, the instrument showed correlations with variables related to PA barriers and motivations (see Supplementary Table S1), as well as adequate effect sizes and statistical power, alerting us to the need to continue investigating this aspect, as it has not yet been addressed empirically to date.

We also consider as a strength of this study the coordination between both international universities during the actions prior to data collection and subsequently during the completion of the instruments, since a single principal investigator was present to explain the content of the informed consent and address any questions arising among participants in both universities. This provided greater methodological control, with identical instructions and a better understanding of the study's objectives.

### 4.2 Limitations

Although the sample size was relatively close to the calculated population estimation of 270 participants, it was not fully reached because data collection required in-person classroom attendance. This discrepancy indicates that the formula used for sample size estimation does not account for reduced on-site attendance and hybrid learning models, which now allow greater flexibility through the use of digital technologies and the re-evaluation of physical space usage, already established in post–COVID-19 university teaching, with current attendance rates estimated between 44.5% and 76.3% (41, 42). This must be considered in future research.

## 5 Conclusions

Overall, this European sample of medical students, grouped into two PA levels, active and non-active, showed that participants comply with WHO recommendations, as 91.9% were active (30.9% Moderate and 61% High), with US students being significantly more active; 8.1% were inactive, of whom UP-S students accounted for 5.4%.

US and UP-S medical students perceive Lack of time as the main barrier to engaging in more regular PA, primarily due to the high academic demands of the degree, with this barrier being moderately more prevalent in France than in Spain. This requires review by universities in order to provide greater support and opportunities, both within the medical curriculum, and through improved access to university facilities dedicated to promoting healthy PA among students.

The motivations or goals that facilitate physical activity are largely similar in both groups, except for the significantly stronger desire among US medical students to obtain Social Recognition and to increase their Resistance to illness.

The findings also highlight the need for further studies in the European context on the variables analysed in this highly influential and health-determining population, given the marked scarcity of studies in the reference literature.

The ordinal regression analysis of the variables predicting physical activity (PA) level yielded the following conclusions:

- University/Country: Students in Seville are more likely to exhibit higher levels of physical activity than those in Paris.
- Gender: Men are nearly twice as likely as women to achieve higher levels of physical activity.
- Academic year: Advancing academic year is associated with a greater likelihood of higher levels of physical activity.
- Fatigue/laziness: Substantially reduces the likelihood of engaging in higher levels of physical activity.
- Obligations/lack of time: Significantly reduces the likelihood of attaining high levels of physical activity.
- Social recognition: Moderately increases the likelihood of higher physical activity.
- Skill development: Considerably increases the likelihood of higher levels of physical activity.
- Age: Does not have a statistically significant effect on physical activity.

## Data Availability

The original contributions presented in the study are included in the article/Supplementary Material, further inquiries can be directed to the corresponding author.

## Supplementary material

Supplementary Table S1 Kendall's Tau-b non-parametric correlations between variables

Supplementary Table S2 Ordinal regression (cumulative logit model) to predict the level of physical activity (IPAQ PA Level).

Supplementary Table S3 Practical implications derived from the results.
