# Supplementary material for: Barriers, motivations and physical activity among medical students: a comparative study between the University of Seville (Spain) and Paris-Saclay University (France)
Source: Front Sports Act Living. 2026 Jun 29;8:1795740. doi: 10.3389/fspor.2026.1795740 (PMC13359588; doi:10.3389/fspor.2026.1795740)
Supplement: Supplementary Table S1 — Kendall's Tau-b non-parametric correlations between variables [file Table1.pdf]

Supplementary Material

Supplementary Table S1. Kendall's Tau-b non-parametric correlations between variables

Variables		(1)	(2)	(3)	(4)	(5)	(6)	(7)	(8)	(9)	(10)	(11)	(12)	(13)	(14)	(15)	(16)	(17)	(18)
(1) Country	Correl. Coef.	1																	
	Sig.	-																	
(2) Age	Correl. Coef.	.273**	1																
	Sig.	.000	-																
(3) Gender	Correl. Coef.	.143*	.035	1															
	Sig.	.033	.570	-															
(4) Academic year	Correl. Coef.	.722**	.569**	.200**	1														
	Sig.	.000	.000	.003	-														
(5) Distance to gym	Correl. Coef.	-.060	-.078	.043	-.083	1													
	Sig.	.355	.188	.503	.196	-													
(6) Distance to University	Correl. Coef.	.038	-.026	-.012	-.041	.126*	1												
	Sig.	.542	.650	.849	.511	.033	-												
(7) Distance to leisure site	Correl. Coef.	-.164**	-.084	-.055	-.163**	.191**	.297**	1											
	Sig.	.008	.138	.368	.008	.001	.000	-											
(8) IPAQ PA Level	Correl. Coef.	.280**	-.001	.251**	.174**	.037	-.011	.001	1										
	Sig.	.000	.984	.000	.007	.553	.852	.990	-										
(9) Body Image/ Phys. social anxiet	Correl. Coef.	-.053	-.133*	-.085	-.105	-.018	-.001	-.034	-.099	1									
	Sig.	.362	.014	.149	.074	.744	.979	.522	.081	-									
(10) Fatigue/ Laziness	Correl. Coef.	-.256**	-.125*	-.173**	-.252**	.020	.030	.012	-.283**	.405**	1								
	Sig.	.000	.015	.002	.000	.711	.553	.806	.000	.000	-								
(11) Obligations/ Lack of time	Correl. Coef.	-.024	-.002	-.146*	-.022	-.015	.052	-.038	-.276**	.066	.246**	1							
	Sig.	.668	.964	.011	.702	.777	.318	.472	.000	.184	.000	-							

Variables		(1)	(2)	(3)	(4)	(5)	(6)	(7)	(8)	(9)	(10)	(11)	(12)	(13)	(14)	(15)	(16)	(17)	(18)
(12) Environment/ Facilities	Correl. Coef.	-.185**	-.141**	-.062	-.189**	.201**	-.022	-.003	-.026	.296**	.305**	.029	1						
	Sig.	.002	.009	.292	.001	.000	.681	.952	.652	.000	.000	.562	-						
(13) Total ABPEF score	Correl. Coef.	-.182**	-.125*	-.170**	-.194**	.058	.049	-.011	-.262**	.509**	.693**	.415**	.438**	1					
	Sig.	.001	.014	.002	.000	.268	.330	.833	.000	.000	.000	.000	.000	-					
(14) Social Affiliation	Correl. Coef.	-.005	.013	.069	.049	.094	.007	.051	.127*	.025	.003	-.089	.214**	.037	1				
	Sig.	.934	.809	.217	.380	.082	.897	.324	.020	.605	.942	.063	.000	.417	-				
(15) Image	Correl. Coef.	-.007	.017	.111	-.001	-.042	-.021	-.072	-.010	.082	.029	.019	.049	.072	.073	1			
	Sig.	.895	.747	.050	.992	.440	.680	.164	.861	.099	.535	.696	.323	.119	.125	-			
(16) Health Management	Correl. Coef.	.089	.069	-.124*	.050	-.111*	.069	-.016	-.078	-.026	.025	.100*	.090	.066	.121*	.102*	1		
	Sig.	.124	.195	.031	.389	.046	.196	.759	.162	.602	.598	.041	.074	.160	.012	.036	-		
(17) Social Recognition	Correl. Coef.	.134*	.045	.217**	.142*	.085	-.059	-.044	.118*	.142**	.003	-.088	.088	.050	.194**	.413**	.017	1	
	Sig.	.018	.387	.000	.012	.120	.260	.401	.031	.004	.949	.069	.074	.283	.000	.000	.725	-	
(18) Skills Development	Correl. Coef.	.034	-.054	.061	-.013	.113*	-.054	-.003	.235**	-.090	-.169**	-.116*	.079	-.127**	.343**	.056	.236**	.107*	1
	Sig.	.551	.296	.278	.814	.036	.297	.946	.000	.068	.000	.016	.109	.006	.000	.237	.000	.025	-

*The correlation is significant at the .05 level (two-tailed)

**The correlation is significant at the .01 level (two-tailed)
