# Supplementary material for: Barriers, motivations and physical activity among medical students: a comparative study between the University of Seville (Spain) and Paris-Saclay University (France)
Source: Front Sports Act Living. 2026 Jun 29;8:1795740. doi: 10.3389/fspor.2026.1795740 (PMC13359588; doi:10.3389/fspor.2026.1795740)
Supplement: Supplementary Table S2 — Ordinal regression (cumulative logit model) to predict the level of physical activity (IPAQ PA Level). [file Table2.pdf]

Supplementary Material

Supplementary Table S2. Ordinal Regression (Cumulative Logit Model) predicting Physical Activity Level (IPAQ PA Level)

Predictor	β	SE	OR	95% CI OR	p
University/Country (1= UP-S, 2= US)	0.84	0.21	2.32	1.53 – 3.52	
Gender (1= Female, 2= Male)	0.71	0.24	2.03	1.27 – 3.24	.003
Academic Year	0.26	0.10	1.30	1.06 – 1.60	.012
Fatigue / Laziness	–0.91	0.18	0.40	0.28 – 0.58	
Obligations / Lack of time	–0.63	0.20	0.53	0.36 – 0.79	.002
Social Recognition	0.29	0.14	1.34	1.02 – 1.77	.036
Skills Development	0.52	0.17	1.68	1.21 – 2.34	.002
Age	–0.01	0.03	0.99	0.94 – 1.05	.842
Intercept 1 (Low → Medium/High)	–0.41	0.32	—	—	.198
Intercept 2 (Low/Medium → High)	1.12	0.34	—	—	.001

β = log-odds; SE = standard errors; OR = odds ratio; CI = confidence interval; p = p-values. Intercept= thresholds. Ordinal logit model (cumulative proportional odds). In ordinal regression models, intercepts (also referred to as thresholds) represent the cut-off points between categories of the dependent variable. Intercept 1 (Low → Medium/High) corresponds to the boundary separating the 'Low' category from the combined 'Medium' and 'High' categories. Intercept 2 (Low/Medium → High) marks the cut-off between the 'Low/Medium' and 'High' categories. These values are not substantively interpreted but define the thresholds required to estimate cumulative probabilities at each IPAQ level within the ordinal logit model.
