# Supplementary material for: Barriers, motivations and physical activity among medical students: a comparative study between the University of Seville (Spain) and Paris-Saclay University (France)
Source: Front Sports Act Living. 2026 Jun 29;8:1795740. doi: 10.3389/fspor.2026.1795740 (PMC13359588; doi:10.3389/fspor.2026.1795740)
Supplement: Supplementary Table S3 — Practical implications derived from the results. [file Table3.pdf]

Supplementary Material

Supplementary Table S3. Practical implications derived from the results.

Instrument	Main finding	Practical implication	Recent theoretical underpinning
Daily Distances			
	Seville is closer to leisure locations and presents more balanced distances to the university; Paris shows more extreme distances.	Improve accessibility to sports facilities, active routes and recreational spaces near campus, especially in Paris.	The physical environment determines physical activity and active mobility (Sallis et al., 2021; Smith et al., 2022).
IPAQ – Vigorous activity (days and minutes)			
	Seville devotes more days and minutes to vigorous activity (d = 0.63 and d = 0.28).	Provide accessible vigorous activity programmes (HIIT, running groups, cycling) and promote their integration into university routines.	Vigorous activity improves health and well-being in university students (Keating et al., 2020).
IPAQ – Moderate activity (days and minutes)			
	Seville accumulates more days and minutes of moderate activity (d ≈ 0.42–0.43).	Implement accessible moderate activities (dance, urban hiking, functional circuits) and active breaks during classes.	Regular moderate activity reduces stress and improves academic performance (Smith et al., 2022).
IPAQ – Walking / Light activity			
	Seville reports more days and minutes walking (d = 0.25–0.38).	Promote active commuting: safe routes, bicycle parking, and active mobility campaigns.	Active mobility is key in young adults (Sallis et al., 2021).

IPAQ – Sitting time			
	Seville reports less sitting time (d = 0.36).	Reduce sedentary behaviour through active breaks, flexible furniture and short activities between classes.	Prolonged sedentary behaviour affects physical and mental health (Smith et al., 2022).
IPAQ – Total physical activity level			
	Seville shows a higher overall level of physical activity (d = 0.56).	Design comprehensive physical activity promotion programmes in Paris, combining active mobility, structured exercise and recreational activities.	Supportive environments increase total activity levels (Keating et al., 2020).
ABPEF – Fatigue/Laziness			
	Paris reports higher fatigue as a barrier (d = 0.65).	Implement sleep hygiene, stress management and micro-exercise sessions for fatigued students.	Fatigue inhibits exercise practice; exercise reduces fatigue (Kwan et al., 2020; Maher et al., 2021).
ABPEF – Other barriers			
	Very small effects; similar barriers between cities.	Cross-cutting interventions focused on health benefits, well-being and active habits.	Common barriers require universal strategies (Teixeira et al., 2020).
GCEQ – Social recognition			
	Seville scores higher in social recognition (d = 0.38).	Design group-based interventions, collective challenges and interfaculty activities leveraging social motivation.	Social support increases adherence to exercise (Laird et al., 2021).
GCEQ – Other motives			
	Null or very small effects.	Interventions applicable in both contexts, focused on health, well-being and autonomous motivation.	Autonomous motivation predicts adherence (Ryan & Deci, 2020).
